# Tissue and circulating biomarkers of benefit to immunotherapy in extensive-stage small cell lung cancer patients

**DOI:** 10.3389/fimmu.2024.1308109

**Published:** 2024-01-29

**Authors:** Martina Lorenzi, Maria Vittoria Resi, Laura Bonanno, Stefano Frega, Alessandro Dal Maso, Alessandra Ferro, Valentina Guarneri, Giulia Pasello

**Affiliations:** ^1^ Department of Medical Oncology, Santa Chiara Hospital, Trento, Italy; ^2^ Division of Medical Oncology 2, Veneto Institute of Oncology - IRCCS, Padova, Italy; ^3^ Department of Surgery, Oncology, and Gastroenterology, University of Padova, Padova, Italy

**Keywords:** ES-SCLC, extensive stage small-cell lung cancer, liquid biopsy, immune checkpoint inhibitors (ICIs), predictive role, biomarkers, tumor immune microenvironment

## Abstract

Extensive stage-Small-Cell Lung Cancer (ES-SCLC) is an aggressive cancer with dismal prognosis. The addition of immune-checkpoint inhibitors (ICIs) to platinum-based chemotherapy have been consistently demonstrated to improve outcomes and survival, becoming the new standard in first – line treatment of ES-SCLC patients. However, despite positive results reported in the pivotal trials, longer benefit appears evident only for a selected group of patients. Several predictive biomarkers have been studied so far but the prospective identification of patients more likely to experience better outcome seems to be challenging in SCLC. Indeed, classical immune predictive biomarkers as PD-L1 and tumor mutational burden (TMB) seem not to correlate with outcomes. Recently, a new molecular classification of SCLC based on differential expression of genes associated with specific clinical behaviors and therapeutic vulnerability have been presented suggesting a new field to be investigated. Despite the achievements, these studies focused mainly on inter-tumoral heterogeneity, limiting the exploration of intra-tumoral heterogeneity and cell to cell interactions. New analysis methods are ongoing in order to explore subtypes plasticity. Analysis on single biopsies cannot catch the whole genomic profile and dynamic change of disease over time and during treatment. Moreover, the availability of tissue for translational research is limited due to the low proportion of patients undergoing surgery. In this context, liquid biopsy is a promising tool to detect reliable predictive biomarkers. Here, we reviewed the current available data on predictive role of tissue and liquid biomarkers in ES-SCLC patients receiving ICIs. We assessed latest results in terms of predictive and prognostic value of gene expression profiling in SCLC. Finally, we explored the role of liquid biopsy as a tool to monitor SCLC patients over time.

## Introduction

Small cell lung cancer (SCLC) is an aggressive disease strongly associated with exposure to tobacco carcinogens with a dismal prognosis and occurs in approximately 15% of lung cancer patients (1, 2). It is a high-grade neuroendocrine carcinoma arising predominantly in current or former smokers, marked by an exceptionally high proliferative rate, strong predilection for early metastasis and poor prognosis. Usually diagnosed in stage IV or III not susceptible of radical intent (VIII edition of the TNM staging system) (generally defined extensive-stage disease (ES) according to the Veterans Administration Lung Study Group’s, VALSG two stage classification) (3), systemic therapy can palliate symptoms and prolong survival, although long-term survival is rare (4).

A platinum agent (cisplatin or carboplatin) together with etoposide have been the standard of care for over 40 years. Despite high initial sensitivity to chemotherapy, responses are transient with a median progression-free survival (PFS) of 5-6 months and a median overall survival (OS) of approximatively 9-10 months (5). At progression, rechallenge with platinum-based doublet or single-agent chemotherapy (CT) with topotecan or lurbinectedin, are the current available options (6, 7). Recently, new standards of care were established in first-line therapy of ES-SCLC based on the positive results of many randomized controlled trials (RCTs) which reported the superiority of the addition of immune checkpoint inhibitors (ICIs) to standard platinum-based CT. Currently, based on the pivotal trials results, IMpower133 and CASPIAN, only the anti-PD-L1 antibodies atezolizumab and durvalumab, granted approval in front line setting (8, 9). However, longer benefit appears evident only for a selected group of patients (8–13). Several predictive biomarkers have been studied so far but the prospective identification of patients more likely to experience better outcome seems to be challenging in SCLC.

**Figure 1:**
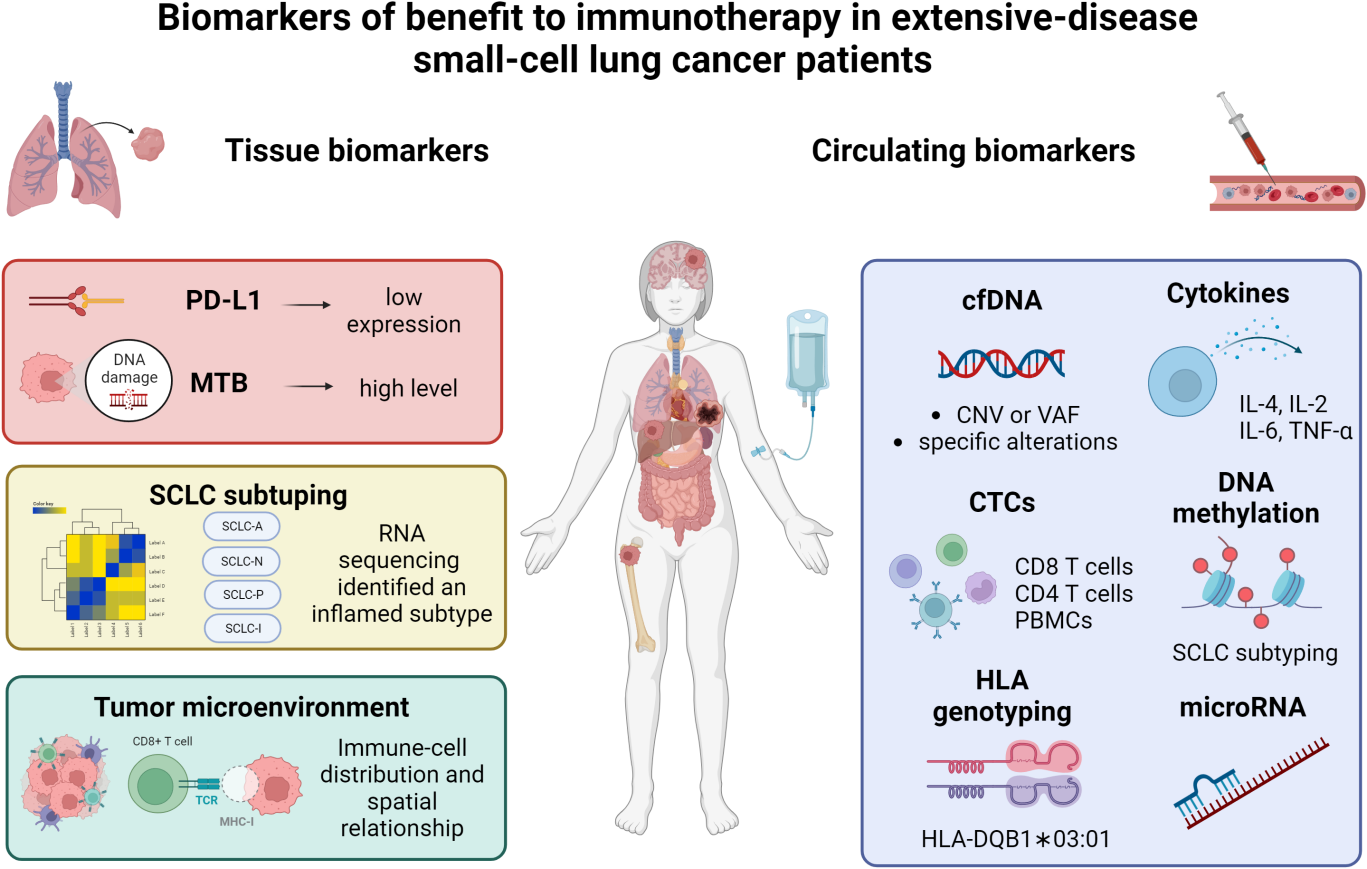
Summary of the current data on biomarkers of benefit to immunotherapy in extensive-stage small-cell lung cancer patients. PD-L1, programmed death-ligand 1; MTB, mutational tumor burden; SCLC, small-cell lung cancer; cfDNA, circulating free DNA; CTCs, circulating tumor cells; PBMCs, peripheral blood mononuclear cells; HLA, human leukocyte antigen. Created with biorender.com.

In this article, we briefly summarized currently available data on immunotherapy in SCLC and reviewed the predictive role of tissue and circulating biomarkers in SCLC patients receiving ICIs; additionally, we explored the role of liquid biopsy as a tool to monitor SCLC patients over time. **Figure 1** summarized the principal biomarkers investigated in ES-SCLC receiving ICIs.

## Materials and methods

**Figure 2:**
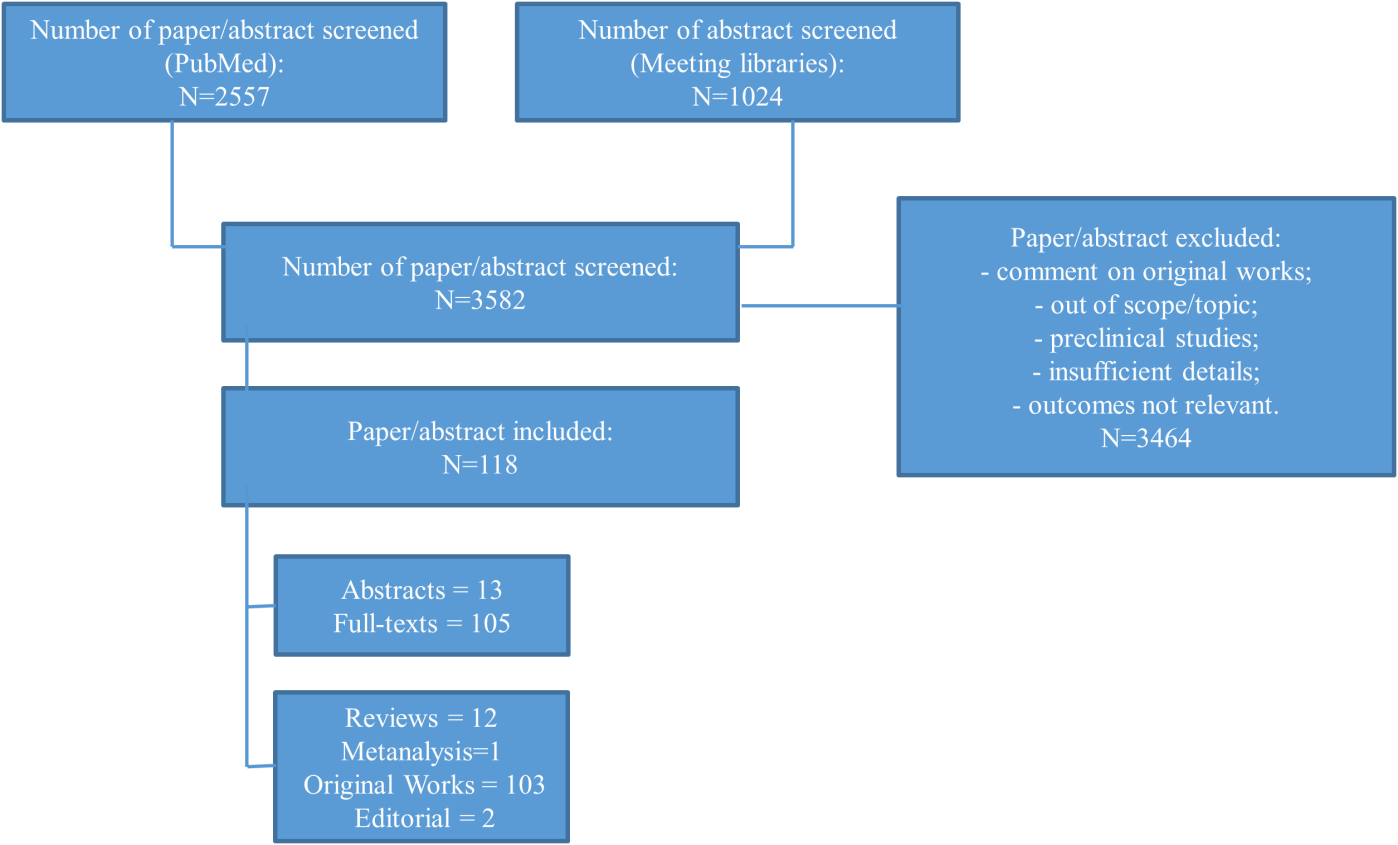
Diagram reporting the flow of information through the different phases of the review.

For this narrative review, we performed an electronic search of the literature using PubMed (September, 8^th^, 2023). We included the key terms “SCLC AND ICIs”, “SCLC AND BIOMARKERS” or “SCLC AND PD-L1”, “SCLC AND TMB”, “SCLC AND tumor microenvironment” and “SCLC AND liquid biopsy”. Based on abstract, appropriate articles were selected. Based on the full-text articles, we excluded studies without relevant information or outside the aim of the present work. The meeting libraries of the largest oncological conferences, in particular from World Conference on Lung Cancer, European Lung Cancer Congress, American Society for Clinical Oncology (ASCO) and European Society of Medical Oncology (ESMO) were also checked (**Figure 2**).

### Immune checkpoint inhibitors as single agent in small-cell lung cancer

ICIs in SCLC have been initially studied in pretreated patients, in the wave of the excellent results obtain in other malignancies, in particular non-small cell lung cancers (NSCLC). The rationale for the use of immunotherapy was the theoretical idea that the strong correlation with cigarette smoking would implies a potentially high tumor mutational burden (TMB) as well as high neoantigen diversity. However, data on ICIs in SCLC suggested only modest responses to single-agent immunotherapy and limited clinical benefit, suggesting a not completed knowledge of the biology of the disease. Indeed, overall response rates (ORRs) are low, ranging between 10% and 35%, and median OS is comprised between 7 and 9 months in the selected population of RCTs. **Table 1** reported pertinent studies in relapsed/refractory SCLC patients.

**Table 1:** Studies concerning immune-checkpoint inhibitors in relapsed/refractory SCLC patients.

Trial	Phase	Population	Primary endpoint	N. of patients	Setting	Treatment arms	mPFS (months)	mOS (months)	mDoR (months)	ORR %
**CheckMate 032 (** 14, 15)	I/II basket	Relapsed ES-SCLC	ORR	216 (98 vs 61 vs 54)	≥3rd	Nivolumab (3 mg/kg q2w) vs Nivolumab (1 mg/kg q3w) plus Ipilimumab (3 mg/kg q3w) vs Nivolumab (3 mg/kg q3w) + Ipilimumab (1 mg/kg q3w)	1.4 vs 2.6 vs 1.4 (p value NR)	4.4 vs 7.7 vs 6.0 (p value NR)	NR vs 7.7 vs 4.4 (p value NR)	10.0 vs 23 vs 19 (p value 0.03)
**CheckMate 331 (** 16)	III	Relapsed ES-SCLC	OS	569 (284 vs 285	≥2nd	Nivolumab (240 mg q2w) vs topotecan (1.5 mg/m2 iv q3w or 2.3 mg/m2 os dd1-5 q3w) or amrubicin (40 mg/m2 dd1-3 q3w)	1.4 vs 3.8 (p value NR)	7.5 vs 8.4 (p value 0.11)	8.3 vs 4.5 (p value NR)	13.7 vs 16.5 (p value NR)
**KEYNOTE-028 (** 17)	Ib (single arm)	Relapsed ES-SCLC, PD-L1 CPS ≥ 1	Safety, tolerability and ORR	24	≥1st (included adj or neoadj therapies)	Pembrolizumab (10 mg/kg q2w)	1.9 (p value NR)	9.7 (p value NR)	NR	33.3 (p value NR)
**KEYNOTE-158 (** 18)	II (single arm)	Relapsed ES-SCLC	ORR	107	≥2nd	Pembrolizumab (200 mg q3w)	2 (p value NR)	9.1 (p value NR)	NR	18.7 (p value NR)
**IFCT-1603 (** 19)	II	Relapsed ES-SCLC	ORR at 6 weeks	64 (43 vs 20)	2nd	Atezolizumab (1200 mg q3w) vs CT (topotecan or re-induction of initial CT)	1.4 vs 4.3 (p value 0.004)	9.5 vs 8.7 (p value 0.60)	NR	6-month - 2.3 vs 10 (p value NR)
**Malhotra et al. (** 20)	I/II	Relapsed ES-SCLC	Safety, tolerability	42 (30 vs 12)	≥2nd	Rova-T [anti DLL3] (0.3 mg/kg q6w for 2 cycles) plus Nivolumab (360 mg q3w for 2 cycles) followed by Nivolumab (480 mg q4w) vs Rova-T (0.3 mg/kg q6w for 2 cycles) plus Nivolumab (1 mg/kg q3w for 4 cycles) plus Ipilimumab (1 mg/kg q3w for 4 cycles) followed by Nivolumab (480 mg q4w)	4.8 vs 4.1 (p value NR)	7.4 vs 11.0 (p value NR)	3.8 vs 3.3 (p value NR)	27.6 vs 36.4 (p value NR)
**PASSION trial (** 21)	II	Relapsed ES-SCLC	ORR	47	≥2nd	Camrelizumab 200 mg q2w plus oral apatinib 375 mg once daily	3.6 (p value NR)	8.4 (p value NR)	NR	34.0 (p value NR)
**Akamatsu et al. (** 22)	II	Relapsed ES-SCLC	ORR	25	≥2nd	Amrubicin (40 mg/m2) on days 1 to 3 plus Pembrolizumab (200 mg, flat dose) q3w	4.0 (p value NR)	10 (p value NR)	4.3 (p value NR)	52 (p value NR)
**Ares et al. (** 23)	I	Relapsed ES-SCLC	Safety, tolerability	107	≥2nd	Tarlatamab [anti DDL3 and anti CD3] (from 0.003 mg to 100 mg q2w)	3.7 (p value NR)	13.2 (p value NR)	12.3 (p value NR)	23.4 (p value NR)
**Ahn et al. (** 24)	II	Relapsed ES-SCLC	ORR	220	≥2nd	Tarlatamab (10 mg q2w vs 100 mg q2w)	4.9 vs 3.9 (p value NR)	NR	NR	40 vs 32 (p value NR)

ES-SCLC, extensive stage small-cell lung cancer; ORR, overall response rate; N, number; vs, versus; mPFS, median progression-free survival; mOS, median overall survival; mDoR, median duration of response; adj, adjuvant; neoadj, neoadjuvant; CT, chemotherapy; qN (2,3,4,6) w, once every N (2,3,4,6) weeks; NR, not reported.

The early phase CheckMate-032 study showed clinically meaningful activity and acceptable safety profile of nivolumab alone or in combination with ipilimumab in previously treated SCLC patients (14, 15). Unfortunately, the subsequent phase III study, comparing nivolumab with CT (topotecan or amrubicin), failed to meet its OS primary endpoint in relapsed SCLC patients (16). Concerning the anti-PD1 pembrolizumab, the phase 1b KEYNOTE-028 trial and the phase II KEYNOTE-158 trial, reported encouraging ORR with the ICI in ES-SCLC, especially in patients with PD-L1–positive tumors (17, 18). However, no OS benefit has been demonstrated. Similarly, the phase II IFCT-1603 trial failed to show a significant role for single-agent atezolizumab in relapsed SCLC cases (19). Given limited efficacy of ICIs alone, combination with other drugs with different mechanism of action was tested. The phase II PASSION trial evaluated the combination of an ICI, camrelizumab, with the anti-VEGFR apatinib, in patients with refractory ES-SCLC reporting potential antitumor effect and manageable safety profile (21). Akamatsu et al. evaluated safety and efficacy of amrubicin plus pembrolizumab (22). The study met its primary endpoint of ORR. Finally, durable objective responses and promising survival outcomes emerged from the phase II DeLLphi-301 trial, investigating the safety and activity of tarlatamab, a bispecific T-cell engager immunotherapy targeting delta-like ligand 3 and CD3 (23, 24).

### Immune checkpoint inhibitors in combination with chemotherapy

Due to the modest and heterogenous response to immunotherapy, evidence suggests that combination of chemotherapy and immunotherapy might improve cancer patient survival compared to monotherapy. No study including immunotherapy as a single-agent approach in the first-line setting was performed due to the aggressive behavior, the rapid growth and the response rate expected to first-line CT.

Recently, the addition of ICIs to platinum-based CT have been consistently demonstrated to improve outcomes and survival in first-line treatment of ES-SCLC patients. **Table 2** reported first-line trial with CT plus ICIs published so far.

**Table 2:** Studies concerning immune checkpoint inhibitors in untreated ES-SCLC patients.

Trial		Phase	Population		Primary endpoint		Number of patients	Setting	Treatment arms	mPFS (months)	mOS (months)	mDoR (months)	ORR %
**IMpower133 (** 9)		III	Untreated ES-SCLC		PFS and OS		403 (201 vs 202)	1^st^ line	Atezolizumab (1200 mg q3w) plus CT (carboplatin AUC5 and etoposide 100 mg/mq, dd1-3 q3w) vs CT (carboplatin AUC5 and etoposide 100 mg/mq, dd1-3 q3w)	5.2 vs 4.3 (p value = 0.02)	12.3 vs 10.3 (p value = 0.007)	4.2 vs 3.9 (p value NR)	60.2 vs 64.4 (p value NR)
**CASPIAN (** 8, 10)		III	Untreated ES-SCLC		OS		537 (268 vs 269)	1^st^ line	Durvalumab (1500 mg q3w) plus CT (etoposide 80–100 mg/m^2^ dd 1–3 plus carboplatin AUC5-6 or cisplatin 75–80 mg/m2 q3w) vs Durvalumab (1500 mg q3w) plus Tremelimumab (75 mg q3w) plus CT vs CT	5.1 vs 4.9 vs 5.4 (p value NR)	12.9 vs 10.4 vs 10.5 (p value = 0.045; p value = 0.0032)	5.1 vs 5.2 vs 5.1 (p value NR)	68 vs 58 vs 58 (p value NR)
**KEYNOTE-604 (** 13)		III	Untreated ES-SCLC		PFS and OS		453 (228 vs 225)	1^st^ line	Pembrolizumab (200 mg q3w) plus CT (etoposide 100 mg/m2 dd 1-3 q3w plus carboplatin AUC5 or cisplatin 75 mg/m2 q3w) vs CT	4.5 vs 4.3 (p value = 0.0023)	10.8 vs 9.7 (p value = 0.164)	4.2 vs 3.7 (p value NR)	70.6 vs 61.8 (p value NR)
**CAPSTONE-1 (** 11)		III	Untreated ES-SCLC		OS		462 (230 vs 232)	1^st^ line	Adremelimab (20 mg/kg q3w) plus CT (etoposide 100 mg/m2 dd 1-3 q3w plus carboplatin AUC5 q3w) vs CT	5.8 vs 5.6 (p value < 0.0001)	15.3 vs 12.8 (p value = 0.0017)	5.6 vs 4.6 (p value NR)	70.4 vs 65.9 (p value NR)
**ASTRUM-005 (** 12)		III	Untreated ES-SCLC		OS		585 (389 vs 196)	1^st^ line	Serplulimab (4.5 mg/kg q3w) plus CT (etoposide 100 mg/m2 dd 1-3 plus carboplatin AUC5 q3w) vs CT	5.7 vs 4.3 (p value NR)	15.4 vs 10.9 (p value < 0.001)	5.6 vs 3.2 (p value NR)	80.2 vs 70.4 (p value NR)
**Arriola E et al; 2016 (** 25)		II	Untreated ES-SCLC		PFS		42	1^st^ line	Ipilimumab (10 mg/kg q3w) plus CT (etoposide 120 mg/m2 iv d1 and 100 mg twice dd2-3 plus carboplatin AUC6 q3w)	6.9 (p value = 0.036)	17.0 (p value = 0.144)	NR	84.8 (p value NR)
**Reck M et al; 2016 (** 26)		III	Untreated ES-SCLC		OS		954 (478 vs 476)	1^st^ line	Ipilimumab (10 mg/kg q3w) plus CT (etoposide 100 mg/m2 dd1-3 q3w plus carboplatin AUC5 or cisplatin 75mg/m2 q3w) vs CT	4.6 vs 4.4 (p value = 0.0161)	11.0 vs 10.9 (p value = 0.3775)	4.0 vs 3.5 (p value NR)	62 vs 62 (p value NR)
**REACTION (** 27)		II	Untreated ES-SCLC after 2 cycles of cis/carboplatin and etoposide with partial or complete response		PFS		119 (58 vs 61)	1^st^ line	Pembrolizumab in combination with platinum-etoposide for 4 cycles then pembrolizumab up to 35 cycles vs 4 cycles of platinum-etoposide	4.7 vs. 5.4 (p value = 0.194)	12.3 vs 10.4 (p value = 0.097)	NR	NR
**CheckMate - 451 (** 28)		II	ES-SCLC after induction CT		OS		834 (279 vs 280 vs 275)	maintenance treatment after 1st line CT	Nivolumab (1 mg/kg q3w) plus ipilimumab (3 mg/Kg q3w) vs Nivolumab (240 mg q2w) vs placebo	1.7 vs 1.9 vs 1.4 (p value NR)	9.2 vs 10.4 vs 9.6 (p value = 0.37)	10.2 vs 11.2 vs 8.1 (p value NR)	9.1 vs 11.5 vs 4.2 (p value NR)
**Perez et al (** 29)		I/II	ES-SCLC after induction CT		Safety/6-months PFS		21	maintenance treatment after 1st line CT	Thoracic RT followed by Nivolumab (1 mg/kg) plus Ipilimumab (3 mg/kg) q3w for 4 cycles followed by Nivolumab 480 mg maintenance	4.4 (p value NR)	11.7 (p value NR)	NR	NR
**Gadgeel et al. (** 30)		II	ES-SCLC after induction CT		PFS		45	maintenance treatment after 1st line CT	Atezolizumab 200 mg q3w	1.4 (p value NR)	9.6 (p value NR)	NR	11%

ES-SCLC, extensive stage small-cell lung cancer; ORR, overall response rate; N, number; vs, versus; mPFS, median progression-free survival; mOS, median overall survival; mDoR, median duration of response; adj, adjuvant; RT, radiotherapy; CT, chemotherapy; qN(2,3,4,6)w, once every N (2,3,4,6) weeks; NR, not reported.

In particular, new standards of care were established in first-line therapy based on two double- blind, phase III RCTs: IMpower133 and CASPIAN (8–10).

IMpower133 trial assessed the efficacy and safety of the combination of atezolizumab with carboplatin plus etoposide followed by atezolizumab maintenance compared to CT alone in treatment-naive patients (9). Results shown a statistically significant improvement in survival reporting a median (m) OS of 12.3 with the combination compared with 10.3 months [Hazard Ratio (HR) 0.70, 95% confidence interval (CI), 0.54-0.91, p = 0.007] with CT and a mPFS, a co-primary endpoint, of 5.2 months (95% CI 4.4-5.6 months) for atezolizumab versus 4.3 months (95% CI 4.2-4.5 months) for placebo (HR 0.77; 95% CI 0.62-0.96; p = 0.017) (9).

CASPIAN is a three-arm RCT evaluating the association of thePD-L1 inhibitor durvalumab, with or without the CTLA-4 inhibitor tremelimumab, and platinum-based chemotherapy followed by durvalumab maintenance compared with CT alone in untreated patients (10). A statistically significant prolongation of OS was reported with the addition of durvalumab to CT, with a median OS of 12.9 months (95% CI 11.3-14.7 months) for durvalumab versus 10.5 months (95% CI 9.3-11.2 months) for CT alone (HR 0.75, 95% CI 0.62–0.91, p= 0.0032). The addition of tremelimumab to durvalumab failed to show any further improvement in outcomes compared with CT (mOS 10.4 months versus 10.5 months, respectively, HR 0.82; 95% CI 0.68–1; *p*= 0.045), with an increased risk of adverse events (8, 10).

### Predictive biomarkers

Looking at the survival curves of ICIs plus CT, the divergence in OS is evident after 6-months of treatment suggesting that only a small proportion of patients benefits from the addition of ICIs. However, no consistent predictive factors have been identified (31). Difficulties in biomarkers detection are related, among others, to the availability of quality and quantity sufficient material to perform molecular analysis and the heterogeneity of the disease, which is related to the high biological plasticity of this malignancy and its ability to adapt to different growth conditions (32).

Here we review the current available data on tissue and circulating biomarkers.

### PD-L1 expression

PD-L1 immunohistochemistry expression have been established as a predictive factor for immunotherapy response in many cancer types, especially in NSCLC (33). PD-L1 expression in SCLC patients seems to be less frequent compared to NSCLC, where is reported to be positive in more than 60% of cases (18, 34), and predominant in the stromal cells, compared to tumor cells (TCs). However, the proportion of PD-L1 positive TCs and immune cells (ICs) in SCLC varies importantly across the studies ranging from 0 to 80% on TCs and from 25 to 54% on ICs, largely due to different cut-off and antibodies applied (17, 21, 35–40).

Despite several studies investigated PD-L1 expression on tumor samples, its predictive role in SCLC patients remains controversial. **Table 3** summarized ICIs results according to PD-L1 populations.

**Table 3:** Studies concerning immune checkpoint inhibitors according PD-L1 expression analysis in ES-SCLC patients.

Trial	Phase	Population	PD-L1 pattern	PD-L1 assay	PD-L1 positivity	Primary endpoint	Setting	Treatment arms	mPFS (months)	mOS (months)	ORR %
**KEYNOTE-028 (** 17)	Ib (single arm)	Relapsed ES-SCLC, PD-L1 CPS ≥ 1	Tumor cells, immune cells and stroma (CPS)	IHC 22C3 clone	31.7% of patients screened	Safety, tolerability and ORR	≥1st	Pembrolizumab	1.9 (p value NR)	9.7 (p value NR)	33.3 (p value NR)
**KEYNOTE-158 (** 18)	II (single arm)	Relapsed ES-SCLC	CPS	IHC 22C3 clone	39%	ORR	≥2nd	Pembrolizumab	2.1 in PD-L1 positive vs 1.9 in PD-L1 negative (p value NR)	14.6 in PD-L1 positive vs 7.7 in PD-L1 negative (p value NR)	35.7% in PD-L1 positive vs 6% in PD-L1 negative (p value NR)
**Akamatsu et al. (** 22)	II	Relapsed ES-SCLC	CPS	IHC 22C3 clone	76%	ORR	≥2nd	Amrubicin plus Pembrolizumab	4.4 in PD-L1 positive vs 3.0 in PD-L1 negative (HR 0.73, 95% CI: 0.25–1.91)	NR	58% in PD-L1 positive vs 33% in PD-L1 negative (p value NR)
**PASSION trial (** 21)	II	Relapsed ES-SCLC	NR	NR	23.4%	ORR	≥2nd	Camrelizumab 200 mg every 2 weeks plus oral apatinib 375 mg once daily	3.6 in PD-L1 positive vs 3.7 in PD-L1 negative (p value NR)	6.6 in PD-L1 positive vs 9.3 in PD-L1 negative (p value NR)	45.5% in PD-L1 positive vs 33.3% in PD-L1 negative
**Gadgeel- et al. (** 30)	II	ES-SCLC after induction CT	Tumor cells and stroma separately (mPS)*	IHC 22C3 Ab	20% (at the stromal interface)	PFS	maintenance treatment after 1st line CT	Atezolizumab	6.5 in PD_1 positive vs 1.3 in PD-L1 negative. (p value NR)	12.8 in PD-L1 positive vs 7.6 in PD-L1 negative (p value NR).	NR
**CheckMate-032 (** 14, 15)	I/II basket	Relapsed ES-SCLC	Tumor cells	IHC Dako 28-8 clone	17%	ORR	≥2nd	Nivolumab vs Nivolumab plus Ipilimumab	NR	NR	NR
**CheckMate-331 (** 16)	III	Relapsed ES-SCLC	Tumor cells and immune cells (CPS)	IHC Dako 28-8 clone	45%	OS	≥2nd	Nivolumab vs topotecan or amrubicin	1.5 with nivolumab vs 4.4 with CT (HR, 1.52, 95% CI, 1.06-2.19) in PD-L1 positive compared with 1.4 with nivolumab vs 4.1 with CT (HR 1.68, 95% CI, 1.23-2.31) in PD-L1 negative	7.0 with nivolumab vs 8.6 with CT (HR 0.96, 95% CI, 0.67-1.38) in PD-L1 positive compared with 7.3 with nivolumab vs 8.1 with CT (HR, 0.91, 95% Cl, 0.66-1.25) in PD-L1 negative	NR
**IFCT-1603 (** 19)	II	Relapsed ES-SCLC	Tumor cells, immune cells and composite score)	IHC SP142 clone	1.8% on TCs and 30% on ICs	ORR at 6 weeks	2nd	Atezolizumab vs CT (topotecan or re-induction of initial CT)	Reported no statistically difference.	Reported no statistically difference.	6-month DCR– 0% in PD-L1 IC positive vs 25% in PD-L1 IC negative (p=0.31)
**IMpower133 (** 9)	III	Untreated ES-SCLC	Tumor cells or immune cells	IHC SP263 clone	52.6% on TCs or ICs	PFS and OS	1^st^ line	Atezolizumab plus CT (carboplatin and etoposide) vs CT (carboplatin and etoposide)	NR	10.2 with ICIs plus CT vs 8.3 with CT (HR, 0.51; 95% CI, 0.30–0.89) in PD-L1 negative; 21.6 vs 9.2; (HR, 0.60; 95% CI, 0.25 -1.46) in PD-L1 >5%	75.0% with ICI plus CT vs 62.2% with CT in PD-L1 negative; 52.8% with ICI plus CT and 69.4% with CT in PD-L1 positive.
**CASPIAN (** 8, 10)	III	Untreated ES-SCLC	Tumor cells or immune cells	IHC SP263 clone	5.1% in TCs; 22.4% on ICs.	OS	1^st^ line	Durvalumab plus CT (etoposide plus carboplatin or cisplatin) vs Durvalumab plus Tremelimumab plus CT vs CT alone	No difference reported	No significant impact of PD-L1 reported (TC, p=0.54; IC, p=0.23).	No difference reported
**KEYNOTE-604 (** 13)	III	Untreated ES-SCLC	CPS	IHC 22C3 clone	40.8%	PFS and OS	1^st^ line	Pembrolizumab plus CT (etoposide plus carboplatin AUC5 or cisplatin) vs CT	Median NR; HR 0.73 (95%CI, 0.54 - 1.01) for PD-L1 negative vs HR 0.68 (95%CI, 0.49 - 0.94) for PD-L1 positive	Median NR; HR 0.80 (95%CI, 0.58 - 1.11) for PD-L1 negative vs HR 0.84, (95%CI, 0.60 - 1.18) in PD-L1 positive	NR
**ASTRUM-005 (** 12)	III	Untreated ES-SCLC	TPS	IHC 22C3 clone	17%	OS	1^st^ line	Serplulimab plus CT (etoposide and carboplatin) vs CT	NR	15.0 with serplulimab vs 10.5 with CT (HR, 0.58, 95% CI, 0.44-0.76) for pts with PD-L1 negative; not reached vs 12.9, (HR,0.92; 95%CI, 0.44-1.89) for PD-L1 positive. Posthoc test for interaction did not show significant interactions.	NR
**CAPSTONE-1 (** 11)	III	Untreated ES-SCLC	TPS	IHC clone, E1L3N	14%	OS	1^st^ line	Adremelimab plus CT (etoposide plus carboplatin) vs CT	Median NR; HR 0·68 (95%CI, 0·54–0·85) in PD-L1 negative. HR 0·70 (95%CI, 0·34–1·45) in PD-L1 positive	Median NR; HR 0·66 (95%CI, 0·52–0·83) for PD-L1 negative; HR 0·72 (95%CI, 0·33–1·59) for PD-L1 positive	NR

*Modified proportion score: mononuclear cells within the tumor cell nests staining for PD-L1 were counted in combination with tumor cells positive for PD-L1.

ES-SCLC, extended stage small-cell lung cancer; ORR, overall response rate; N, number; vs, versus; mPFS, median progression-free survival; mOS, median overall survival; adj, adjuvant; CT, chemotherapy, NR not reported, TPS, tumor proportion score; CPS, combined proportion score; HR, hazard ratio; IHC, immunohistochemistry.

The phase I basket trial KEYNOTE-028, evaluating safety, tolerability and efficacy of pembrolizumab in relapsed SCLC patients, included only PD-L1 positive tumors, defined as membranous PD-L1 expression on TCs, ICs or positive staining in stroma (41). The ORR to pembrolizumab was relatively high, reaching the 33% (41). The more recent single arm phase II KEYNOTE-158 trial, enrolling patients irrespective of PD-L1 status, reported a numerically improvement of ORRs (35.7% vs 6%) and mOS (14.6 months vs 7.7 months) in PD-L1 positive cases (N=42, 39%) compared to negative (N=50, 47%) receiving pembrolizumab as second line treatment (18, 41). Similar results were obtained in a phase II trial of pembrolizumab associated with amrubicin in pretreated SCLC patients (22). Cases with a positive PD-L1 combined positive score (PD-L1 CPS, N=19, 76%), tended to have better efficacy outcomes than those with CPS less than 1% or not assessable (N=6, 24%) in terms of ORR (58% versus 33%) and mPFS (4.4 versus 3.0 months, HR 0.73, 95% CI: 0.25–1.91) (22).

Low levels of PD-L1 expression (23.4%) were found also in PASSION trial, evaluated camrelizumab plus apatinib in pretreated SCLC patient (21). Again, in a *post hoc* subgroup analysis, a numerically higher ORR in PD-L1 positive compared with negative (45.5% vs 33.3%) patients was reported. However, the small sample size and the single arm nature of the study do not permit to clarify the role of this biomarker (21).

In the phase II study by Gadgeel et al., evaluating pembrolizumab maintenance after induction platinum-etoposide CT, only 10% of the evaluable cases had a PD-L1 expression on TCs; whereas 40% stained positive for PD-L1 at the stromal interface (30). In this subgroup, a numerically better outcome in terms of PFS (6.5 vs 1.3 months) and OS (12.8 vs 7.6 months) was reported (30).

These data generate the hypothesis of PD-L1 staining as potential predictive biomarker of response in patients treated with pembrolizumab. However, no consistent data are available for other PD-L1 agents.

The Checkmate 032 trial, evaluating nivolumab with or without ipilimumab, enrolled pretreated SCLC patients regardless of PD-L1–expression status (14, 15). Low positive PD-L1 expression on TCs was reported (17%). The ORR was low with single-agent nivolumab (ORR 10%) and no significant correlation was found between response and PD-L1 status in the pre-planned exploratory analysis, neither for patients receiving nivolumab nor for patients receiving nivolumab plus ipilimumab (14, 15).

In the phase II Checkmate 331 RCT, comparing nivolumab with CT in relapsed SCLC patients, PD-L1 CPS positive tumors were 45% of the evaluable population. The biomarker analysis found comparable outcomes in patients with PD-L1 CPS positive and negative tumors in term of OS and PFS (16). Similar results were reported in the randomized non comparative phase II IFCT-1603 trial of pembrolizumab or CT as second line therapy (19). In particular, only one case (1.8%) was proven positive for PD-L1 on TCs, whereas 30% of cases expressed PD-L1 on ICs. No statistically significant difference in PFS and OS were found (19).

Moving from studies in which the ICIs are used as single agent to combination therapy trials, PD-L1 as biomarker definitively loses his putative predictive role.

In the IMpower133 RCT, only 34% of the intention to treat population was evaluable for biomarker analysis. The study confirmed a low expression of PD-L1since 5.8% of patients stained positive for PD-L1 on TCs and 50.4% on ICs (42, 43). An OS benefit with atezolizumab plus CT compared with CT alone was registered in PD-L1 negative group (mOS 10.2 months vs 8.3 months; HR, 0.51; 95% CI, 0.30–0.89) and in PD-L1 ≥5% subgroup (mOS 21.6 months vs. 9.2 months; HR, 0.60; 95% CI, 0.25 -1.46). Response rate was numerically higher in PD-L1 negative subgroup receiving atezolizumab plus CT compared to CT alone (75.0% and 62.2%); on the contrary in the PD-L1 positive subgroup ORR were numerically higher for the CT arm (52.8% and 69.4%, respectively) (42, 43).

Similarly, in the phase III CASPIAN trial, low levels of PD-L1 expression were reported (44). Of the 51.6% evaluable cases, 94.9% and 77.6% of patients had a negative PD-L1 expression on TCs and ICs, respectively (44). An exploratory analysis conducted by Paz-Ares et al. confirmed that OS benefit with durvalumab plus CT versus CT was similar across PD-L1 subgroups (HR CI 95%, 0.47-0.79); on the contrary, OS benefit with durvalumab plus tremelimumab vs CT alone was greater in PD-L1 positive tumors (45).

Results of the phase III KEYNOTE-604, evaluating pembrolizumab in addition to platinum-based CT in ES-SCLC patients, are similar to other two RCTs concerning PD-L1: 40.8% had PD-L1 CPS ≥ 1% and survival outcomes were similar in participants with PD-L1–positive and PD-L1–negative tumors (13).

Moving to the more recent phase III ASTRUM-005 study, investigating the effect of first-line serplulimab added to CT in first-line setting, data was inconclusive regarding the predictive role of PD-L1, in line with previous studies (12). An imbalanced OS was reported for serplulimab in patients with PD-L1 TPS of less than 1% (mOS 15.0 months in the serplulimab group vs 10.5 months in the placebo group; HR, 0.58, 95% CI, 0.44-0.76) and for patients with PD-L1 TPS ≥ 1% (not reached vs 12.9months, respectively; HR, 0.92, 95% CI, 0.44-1.89) (12). Consistently, the CAPSTONE-1 trial reported a low level of PD-L1 expression in enrolled patients since 86% of cases had a PD-L1 TPS of less than 1% and no evidence of better outcome in the PD-L1 subgroup analysis (11).

In conclusion, despite the initial hypothesis of a predictive role of PD-L1 expression in ES-SCLC patients receiving ICIs coming from in single arm studies, phase III RCTs were not conclusive and PD-L1 status appear to be not suitable in selecting patients who may have benefit from ICIs. A meta-analysis on the role of PD-L1 expression as predictive biomarker of response would be helpful to further clarify this question.

### Tumor mutational burden (TMB)

Besides PD-L1 expression, TMB, defined as the total number of mutations per coding area of a tumor genome, is regarded as a biomarker of the efficacy of ICIs in various cancers (46, 47). SCLCs have a high median TMB, likely related to the tobacco carcinogenesis (48). TMB was largely investigated as a predictive factor in multiple studies including patients with SCLC receiving ICIs.

In the exploratory analysis of the CheckMate 032 trial, Hellmann et colleagues evaluated TMB in pretreatment tissue and paired blood samples (49). Whole exome sequencing (WES) was used to quantify the total number of somatic missense mutations. Patients were grouped in thirds according to tissue TMB value: low, <143 mutations; intermediate, 143–247 mutations; and high, > 248 mutations. The TMB-evaluable population comprises 221 cases of the overall population (N=401, 53%) (49). Patients treated with nivolumab or with nivolumab plus ipilimumab had higher ORRs in the presence of a high TMB level (21.3% and 46.2%, respectively) compared to medium (6.8% and 16%) or low (4.8% and 22.2%) (49). Moreover, TMB was higher in responsive patients compared to non-responsive ones, either with nivolumab alone or the combination. Concerning survival outcomes, numerically longer PFS and OS were reported in the TMB-high group, especially with nivolumab plus ipilimumab. Indeed, median PFS was 1.3, 1.3 and 1.4 months with nivolumab and 1.5, 1.3 and 7.8 months with the combination, in the low, medium and high TMB groups, respectively. Similarly, mOS was 3.1, 3.9 and 5.4 months with single agent ICI and 3.4, 3.6 and 22 months with the combination. In this exploratory analysis, TMB assessed through WES, well correlates with in silico filtering to the 315 genes in the FoundationOne next generation sequencing profile in terms of number of mutation per million bases, supporting the use of FoundationOne CDx assay in clinical practice (49).

Indeed, WES is the “gold standard” for measuring TMB, allowing the detection of somatic mutations within the entire exome. However, this next generation sequencing platform is difficultly feasible in routine testing because of the long turnaround time, the high costs and the need of sufficient tumor tissue for analysis. On the contrary, the gene panels commercially available for TMB [i.e. FoundationOne CDx assay or Memorial Sloan Kettering-Integrated Mutation Profiling of Actionable Cancer Targets (MSK-IMPACT)] have the limits to cover a restricted number of genes (0.80-2.40 Mb, <5% of the total coding area) and to include also intronic sequence but are less expensive and faster. Therefore, the evaluation of the concordance between WES and genes panels gains importance for them routine clinical application (50).

In the phase III CheckMate 331 trial, TMB was studied with the FoundationOne CDx assay. Of all included patients, only 55% was evaluable for TMB and substantially different survivals were reported between TMB-evaluable vs non-evaluable cases precluding any meaningful analysis of efficacy by TMB status (16).

The role of TMB as a biomarker was also studied in the CheckMate 451 trial, evaluating nivolumab alone or in combination with ipilimumab vs placebo as maintenance in patients with ES-SCLC without progression after first-line CT (28). FoundationOne CDx assay was applied and patients grouped in high and low using two prespecified cut-off (10 and 13 mut/Mb). The study did not meet the OS primary endpoint, however in the *post hoc* analysis an improved OS with combination therapy (13.5 months; 95%CI, 9.3 - 21.8; HR, 0.61; 95% CI, 0.39 - 0.949) and a trend toward better OS with nivolumab monotherapy (13.2 months; 95%CI, 10.0 - 17.9; HR, 0.67; 95%CI, 0.45 - 1.01) compared to placebo (9.5 months; 95%CI, 6.2 - 13.5) were reported in patients with TMB-high tumors (>13 mut/Mb). Interestingly, a less stringent TMB cutoff (>10 mut/Mb) failed to show a survival benefit in either group (28).

TMB as predictive biomarker was studied also for pembrolizumab. A higher ORR was registered in the exploratory analysis of the phase II KEYNOTE-158 basket trial in patients with TMB-high status (N=34)compared to TMB-low (N= 42, ORR 29.4% vs 9.8%), using 10 mut/Mb as cut-off (51).

These data seem to suggest a predictive role of high TMB, especially in patients receiving ICIs as combination therapy. However, the exploratory nature of the analysis and the absence of a control arm only generate hypothesis. Unfortunately, the phase III trials failed to confirmed its predictive role in first-line setting.

In the exploratory analysis of the CASPIAN trial presented at ESMO Congress in 2020, tissue TMB, assessed using the FoundationOne CDx platform in 35% of the intention-to-treat population, was not predictive of longer OS for durvalumab with or without tremelimumab compared to CT at different pre-specified cut-off (≥8, ≥10, ≥12 and ≥14 mut/Mb) (52, 53). The absence of an interaction between tissue TMB and OS were confirmed by by Paz-Ares et al. in the final analysis (durvalumab plus CT vs CT, p = 0.916; durvalumab plus tremelimumab plus CT vs CT, p = 0.672) (45).

Similarly, Rudin and colleagues in the exploratory analysis of KEYNOTE-604 trial reported the absence of positive association between TMB, OS (p=0.450) and PFS (p=0.362) in the pembrolizumab plus CT arm (54). On the contrary, a longer OS was obtained in the experimental compared to control arm in TMB low (<175 mut/exome) subgroup (mOS 10.2 months, 95%CI 8.5-14.4; vs 7.7 months, 95%CI 6.6 – 9.3; HR 0.60, 95%CI 0.43-0.85) (54).

Finally, in the IMpower133 trial, assessment of TMB was performed with a blood-based assay (bTMB) (9, 42). Blood-based markers could overcome the challenge of obtaining sufficient tissue from SCLC to perform analyses. At two prespecified cut-offs (10 and 16 mut/Mb), no differences in outcome in terms of PFS and OS were registered from the addition of atezolizumab to CT (42).

In conclusion, the role of TMB is not clear in SCLC since it seems to be a predictive factor for pre-treated SCLC receiving ICIs but not for treatment-naïve patients treated with chemo-immunotherapy. This could be explained with the disability of antigen presentation mechanism through the major histocompatibility complex class I (MHC-I) to present neoantigen to cytotoxic T lymphocyte (CTL). Indeed, patients with upregulation in MHC-I, experienced durable benefit from ICIs in a translational study (55). This hypothesis is supported also by the results of the RNA-sequencing analysis performed by Rudin and colleague on patients participating in the CheckMate 032 study, reporting a correlation between antigen machinery signature and survival (p<0.001) (56).

### Tumor microenvironment (TME)

The tumor microenvironment is a complex network comprising blood vessels, infiltrating inflammatory cells, stromal cells and a variety of associated tissue cells which are created and orchestrated by tumor cells through molecular interactions. Infiltrating immune cells include T lymphocytes (CD3+/CD4+, CD3+/CD8+, Treg FOXP3+), dendritic cells, B lymphocytes (CD 20+), macrophages, also known as tumor- associated macrophages (TAMs), leukocytes and rare natural killer (NK) cells (57).

The aggressiveness of SCLC leads to the lack of abundant material for tissue analysis and tumor immune microenvironment description, especially in ES. For this reason, data in TME are limited and particularly difficult to obtain with classical IHC assay. Recently, new technologies have permitted to clarify the tumor heterogeneity and interactions between distinct cell components in the TME, critical to understand the biology of the disease and the susceptibility to specific treatments (32). In particular, single-cell RNA sequencing, performing a molecular characterization of all cell types within a complex population, identifies a highly heterogeneous and immunosuppressive microenvironment in SCLC samples (58).

Tumor infiltrating lymphocytes (TILs) are directly involved in immunologic anti-tumor mechanism and are associated with long term survival in SCLC patients (59). Carvajal-Hausdorf and colleagues reported a significantly lower number of TILs (CD3+, CD8+ and CD20+) in SCLC TME compared to lung adenocarcinoma and squamous carcinoma, defining SCLC as an immune-cold tumor (60). Several studies reported a positive prognostic role for TILs and CD8 + cells but limited data are available on their predictive role (59–62). The *post hoc* analysis of the CheckMate 032 study, assessing CD8+ cells infiltration in pretreatment samples, reported an improved OS in a CD8 positive cohort receiving nivolumab compared to CD8 low cohort (HR = 0.51, 95% CI: 0.27–0.95) and a trend for better OS in patients receiving nivolumab plus ipilimumab. Due to the lack of a control arm, hypothesis of a predictive role of CD8+ cells needs further investigation in large RCTs (56).

In favor of this thesis, the preliminary analysis of a translational study of our group investigating, through multiplex immunofluorescence, the immune cells distribution and spatial relationship within microenvironment as predictive biomarkers of benefit in ES-SCLC patients receiving atezolizumab plus carboplatin and etoposide, was presented at ELCC 2023 (63). In patients analyzed (N=39), data show a positive role of the interaction between CD8+ cells and CD20+ cells on PFS (p = 0.038), TTF (intra-tumoral, p = 0.036) and OS (p = 0.032), and a high percentage of stromal CD163+ closed to CD8+ cells on PFS (p = 0.045) (63). Another retrospective study evaluated the association between the efficacy of atezolizumab plus carboplatin and etoposide and TILs status, in a cohort (N=37) of untreated ES-SCLC. The PFS of patients with TIL_High_ tumor was significantly greater than PFS of patients with TIL_Low_ (PFS 7.3 months, 95% CI, 4.2–10.4 vs. 4.0 months, 95% CI, 2.7–5.3, p<0.001) (64). Similarly, Le Noac’h and colleague conducted a single center retrospective study aiming at characterizing different cell populations through Imaging Mass Cytometry (IMC) in ES-SCLC patients receiving atezolizumab plus platinum-etoposide. A total of 11 out of 20 included cases were evaluable for TME. A positive correlation between percentage of CD4+, CD8+, regulatory T cells (Treg) and longer PFS (p=0,001, p=0,025 and p=0,002 respectively), was reported (65). The small samples size do not permit to make any inference.

Treg cells (detected as FoxP3+ TILs) are generally considered suppressive cells; their expression often results in an immunosuppressive microenvironment and tumor progression in different solid tumors (66, 67). However, FoxP3+ cells are heterogeneous and include also non-suppressive phenotype with an anti-tumor activity (68, 69). In limited-stage SCLCs, two studies reported a positive prognostic role of FoxP3+ cells infiltration on risk of recurrence; in extensive-stage, the positive prognostic role was not demonstrated (39, 70, 71). A positive correlation between intra-tumoral CD4+FoxP3+ and PFS (p=0.004), TTF (p=0.011) and OS (p=0.026) for chemo-immunotherapy was described by our group in the preliminary analysis of the aforementioned translational study (63). Further investigations are needed to confirm their role in SCLC.

Another component of the TME is represented by macrophages. Recent investigations have shown that TAMs can promote tumor development and progression by promoting angiogenesis, matrix remodeling and suppressing adaptive immunity. Macrophages have been classified into two groups according to the phenotype: “classically activated” proinflammatory M1 and “alternatively activated” anti-inflammatory M2 (72). Although macrophages are generally associated with tumor development and progression in many cancer types, some studies on SCLC patients reported contrary results (72, 73). Indeed, Eerola et al. showed a correlation between a high number of intra-tumoral macrophages and a favorable outcome in surgical samples of stage I-IV unselected SCLC patients (74). Similar results were reported by Muppa et al. in resected SCLC patients where CD68-positive macrophages were higher in long-term survivors (59).

In the preliminary analysis of our previously mentioned study, a lower CD163+ M2- polarized macrophages density and ratio on CD8+ cells in the total and tumoral areas were favorably associated with ORR, PFS, TTF and OS thus suggesting a putative predictive role of this component (63).

Finally, myeloid-derived suppressor cells (MDSCs) are a heterogeneous group of immature myeloid cells which produce immunosuppressive signals in the TME (75). Monocytic myeloid-derived suppressor cells (mMDSC) and granulocytic myeloid-derived suppressor cells (gMDSC) signatures evaluated with non-gene expression profile gene sequencing analysis was evaluated in the exploratory analysis of KEYNOTE-604 trial (54). A benefit in PFS was reported by Rudin at ASCO 2023 for pembrolizumab plus CT comparted with CT alone in mMDSC low population (PFS 5.6 months, 95%CI 4.9.7.1; vs 4.2 months, 95%CI, 4.2 -4.9; HR 0.45; 95%CI 0.31-0.64) but not in mMDSC high subgroup. Similar results were obtained for OS in gMDSC group suggesting a potential predictive role of these components (54).

To best of our knowledge, there is a lack of randomized control studies examining the TME as a predictive factor for immunotherapy in SCLC patients. New technologies and promising data existing on this topic should encourage the planning of translational analysis in all new large RCTs.

### Molecular classification and gene expression profiling

A relevant problem in the SCLC knowledge has been for long time the small amounts of material available for histological diagnosis and subsequent research. In recent years, thanks to the improvement in technology, many progress have been made in this field. Epigenetic and transcriptomic analysis revealed a new and previously unappreciated molecular diversity among SCLC. In 2013, Poirer et al. suggested the existence of different subtypes of SCLC on the basis of the analysis of a panel of cell lines for susceptibility to a neuroendocrine cancer-selective oncolytic virus infection in mouse model of SCLC (76). Gene expression profile of these lines then identified two different subtypes of SCLCs characterized by the expression of two transcription factors: achaete-scute homologue 1 (ASCL1) and neurogenic differentiation factor 1 (NeuroD1), involved in the neuroendocrine development of cells of the lung (77). Contemporary, Osborne and colleagues demonstrated that NeuroD1 promotes tumor cell survival and metastasis in a subset of neuroendocrine lung carcinomas through the receptor tyrosine kinase tropomyosin-related kinase B (TrkB) and neural cell adhesion molecule (NCAM) (78). A third transcription factor, POU class 2 homeobox 3 (POU2F3) appear to be expressed in a subtype of SCLC cell lines characterized by low level of both ASCL1 or NeuroD1. POU2F3 is expressed exclusively in variant SCLC tumors that lack expression of neuroendocrine markers and instead express markers of a chemosensory lineage known as tuft cells (79). Despite the identification of this third marker, some SCLC remain unclassifiable. Rudin et al. proposed the transcription factor *YAP1*, a regulator of transcription activated by the HIPPO growth signaling pathway (80). However, subsequent immunohistochemical analysis failed to confirm a unique YAP1 subtype in the patient cohort of SCLC tested (81).

Recently, Gay et al. proposed a classification of SCLC based on the expression of the aforementioned transcription factors: high ASCL1 (SCLC-A) or high NEUROD1 (SCLC-N), POU2F3 (SCLC-P) subgroup and a distinct group of SCLC tumors with lower expression of all three transcription factor signatures (82). The fourth subtype was characterized by the expression of inflammatory genes included numerous immune checkpoints and human leukocyte antigens (HLAs). Thus, this subtype was designated SCLC-inflamed, or SCLC-I (82).

These subtypes are characterized by a differential expression of genes, or rather different gene signatures. Neuroendocrine genes (like the chromogranin marker) are expressed in SCLC-A and SCLC-N; on the contrary, the RE1 Silencing Transcription Factor (REST), a repressor of neuroendocrine genes, is higher in SCLC-I and -P. SCLC-I appeared to be the most mesenchymal. The absolute number of several immune cell populations were markedly increased in SCLC-I, including T-cells, NK cells, and macrophages. Also, HLA, immune checkpoints (PD-1, PD-L1, CTLA4, CD38, ID O 1, TIGIT, VISTA, ICOS, and LAG3) and chemokine (CCL5 and CXCL10), are overexpressed in SCLC-I compared to others, again supporting an inflamed microenvironment (82). Since data came from LS-SCLC patients, analysis was subsequently performed on the ES-SCLC population of the pivotal IMpower133 trial. The distribution of subtypes was: SCLC-A -51%, SCLC-N - 23%, SCLC-I – 18%, SCLC-P – 7%.

In a *post-hoc* analysis of OS of the trial, a trend toward benefit of CT plus atezolizumab is presents across all four subtypes. However, the best OS benefit is evident in SCLC-I subgroup receiving CT plus atezolizumab (mOS for SCLC-I of 18.2 months in the experimental arm, compared to 10.4 months in the control arm), suggesting that SCLC-I subtype is not a prognostic marker (82).

SCLC subtypes and related outcomes were also studied in a cohort of patients included in the CheckMate 032 trial (56). Tumors were classified in four subgroups on the basis of the expression of genes encoding for one of the transcription factors ASCL1, NEUROD1, POU2F3, and YAP1. No significant correlation was identified for each subgroup with outcome, nor comparing neuroendocrine ones (SCLC-A and SCLC-N) with non-neuroendocrine (SCLC-P, SCLC-Y). Of note, a trend toward higher levels of inflammation gene signature in SCLC-Y tumors was reported (56).

The expression of the subtype-defining markers at a protein level has also been investigated through IHC (81). However, data are not conclusive about the predictive role of this classification: the biomarker analysis on the- phase IIIB CANTABRICO study enrolling patients with ES-SCLC receiving durvalumab plus platinum-etoposide as first-line treatment, reported no survival differences between SCLC IHC subtypes in a small cohort of patients (83). Interestingly, 6 out of 9 patients with No-A/N/P tumors were still alive at data cut-off suggesting potentially a better outcome (83).

SCLC transcriptional subtypes analysis is ongoing for patients included in the KEYNOTE-604 trial; however, Rudin et colleague presented at ASCO congress 2023 the role of 18-gene T cell–inflamed gene expression profile (Tcell_inf_GEP) evaluated with RNA sequencing in a cohort of patients included in the study (54). Tcell_inf_GEP were previously studied in the KEYNOTE-028 basket trial using a pan-tumor, 18-gene assay. Its score was found to be higher across tumors achieving ORR and longer PFS (84). In KEYNOTE-604, 70% of the intention-to-treat population, had RNA-seq data available (54). A positive association between higher Tcell_inf_GEP and survival outcomes (OS and PFS) was reported in both treatment groups. Interestingly, duration of response was numerically longer in the pembrolizumab group of patients with high T-cell inflamed signature at baseline. The author concluded that inflamed infiltrate within the tumor may be a prognostic signature associated with benefit regardless the addition of immunotherapy (54).

Investigating the expression 770 immune/cancer-related genes, by the Nanostring® PanCancer IO360 panel, in ES-SCLC treated with carboplatin-etoposide in a real-world cohort of patients, our group reported that, despite absolute CD8+ T-cells and cytotoxic T-cells signature scores were not predictive of better outcome, high cytotoxic T-cells/TILs signature scores ratio was associated with longer PFS (p= 0.006) and TTF (p=0.001). Similarly, high T-cells/TILs ratio (p=0.02), mast cells/TILs (p=0.003) ratio and low macrophages/TILs ratio (p=0.04) were associated with longer PFS (85). These data suggest a role of relative high number of cytotoxic T-cell and low number of macrophages as predictive of better outcome.

In conclusion, data on transcriptomic seems to be a promising field for biomarkers detection in SCLC patients due to the ability to provide big amount of data from relatively low quantity of material. However, RCT powered to demonstrate the role of molecular subtypes or specific inflamed expression gene are urgent needed.

Transcriptomics analysis presents some limitations for future clinical practice application. First of all, actual gene expression profiling platforms need suitable material coming from the small biopsies performed in the diagnostic pathway of SCLC. Secondly, available assays are characterized by a relatively long turnaround time due to multiple parallel runs performing simultaneously in order to reduce costs. Finally, a high qualified staff is needed to performed the analysis and for data interpretation (86).

### Circulating biomarkers in liquid biopsy

Considering the intra-tumoral and inter-tumoral heterogeneity of SCLCs, samples obtained from a single biopsy may not capture the complete molecular profile of the disease. Additionally, temporary heterogeneity, referring to the capacity of SCLC to change during the course of therapy, could be catch only with repeated sampling, not easy feasible in lung cancers (87).

To overcome these limitations, in the last few years there has been an increasing development in liquid biopsy of plasma and other biological fluids. This approach is based on the presence in vessels of several components as circulating tumor cells (CTC), circulating free DNA (cfDNA), circulating tumor RNA (ctRNA), exosomes, microRNA (miRNA) and non-coding RNAs (ncRNAs) that can be used as possible biomarkers (88). Serial liquid biopsies could catch the plasticity and dynamic changes of SCLCs as longitudinal assessment of tumor burden and early resistance mechanisms.

Liquid biopsy has several advantages compared to tissue biopsy. Indeed, it is a minimally invasive, repeatable tool that allows a dynamic evaluation of tumors. Analysis of tumor genetic material could provide both qualitative and quantitative data on tumor-related genomic alterations. Unfortunately, not all patients with advanced disease have detectable tumor material in their bloodstream which can lead to false-negative results and affect the sensitivity of the approach (89).

### Circulating tumor DNA

A recent metanalysis on liquid biopsy in SCLCs, reported a median detection rate of 91% for circulating tumor (ct) DNA, ranging from 49% to 100%, and from 71 to 100% in studies using next-generation sequencing panels (90).

Few data are available on the predictive role of liquid biopsy in ES-SCLC treated with ICIs. Indeed, the studies exploring the role of cfDNA in longitudinal monitoring of the disease included generally a small number of patients treated with CT alone and aimed at identifying the prognostic role. Results suggested an association between quantitative cfDNA level, in terms of variant allele frequency (VAF) or copy number variation (CNV), and outcome, especially OS. Moreover, specific alterations seem to be associated with chemosensitivity, as the *APC* alteration, or chemoresistance (*TP53, ATM*) (91–95).

Concerning immunotherapy, in the ancillary study of the IFCT-1603 trial, a significantly lower disease control rate was reported in SCLC pretreated patients receiving atezolizumab (N=46) or CT (N=22) with detectable circulating mutations, regardless the treatment received (96). This could be explained with higher levels of tumor burden and a worst prognosis, in patients with detectable ctDNA. Moreover, patients with ctDNA abundance (above the median level) experienced a significantly shorter PFS (p<0.01) and OS (p<0.01) compared to others (96). Interestingly, patients with a relatively high cfDNA level reported a trend towards shorter OS with atezolizumab compared to CT; on the contrary, OS benefit was higher for patients treated with atezolizumab in presence of a relatively low cfDNA level (96). Although several limitations, as number of patients included and the exploratory nature of the analysis which reclaim further investigation, this study identified a subgroup of patients more probably to benefit from ICIs (96).

Beside quantitative cfDNA analysis, qualitative cfDNA gene alterations have been investigating in SCLC patients, finding, in a heterogeneous population, genomic aberrations (*SETBP1, PBRM1, ATRX, EP300, ATM, PIK3CA/G, or NOTCH1*) as potential biomarkers of treatment efficacy and prognosis (97).

Sivapalan et al. performed a ctDNA sequencing of serial plasma samples, and combined the analyses of somatic sequence with chromosomal structural alterations in ctDNA, in order to dynamically predict clinical outcomes in a heterogeneous population (N=33) of SCLC patients undergoing systemic therapies (CT, ICIs or ICIs) (98). Results reported a sustained elimination of cell-free tumor load (cfTL) compared to baseline in molecular responders (N=9); initial molecular responses follow by ctDNA recrudescence (N=14) and a pattern persistence of cfTL across timepoints (N=10). In the study, patients with sustained molecular responses performed longer PFS (p< 0.0001) and OS (p= 0.0006) compared with other two groups of molecular response (98).

### Circulating cells

Circulating cells comprise CTCs and immune circulating cells. CTCs are cells directly derived from tumor and find in the bloodstream in small amount; they can be detected using antibody against epithelial protein or exploiting biophysical differences of CTCs from other blood cells. The aforementioned metanalysis reported a median detection rate for CTC of 85%, ranging from 49% to 96%, higher in patients with ES-SCLC compared to LS-SCLC (95% vs. 65%, respectively) (90).

Similarly to ctDNA, studies on CTCs mainly focused on their prognostic role and included small cohorts of heterogeneous patients receiving CT in the pre-immunotherapy era. However, some important information could be addressed. First, high level of CTCs at baseline is a negative prognostic factor for PFS and OS, as confirmed by a meta-analysis of Zhang and collegues (62). Secondly, a clear cut-off has not been identified (99).

Predictive role of CTCs is controversial since some studies reported that a high level of CTCs is associated with worst PFS on CT (100–103), other failed to demonstrate the association (104, 105). However, the change in CTCs level during treatment seems to better predict response to CT (103).

Immune circulating cells, and in particular the dynamic change of peripheral blood mononuclear cells (PBMC) in patients receiving ICIs, are another serum component explored. Perez et al. reported a reduction in peripheral CD8 T cells during treatment with nivolumab and ipilimumab after induction CT and consolidative thoracic radiotherapy in patients who met the primary (6-month PFS) and secondary endpoint (12-months OS) compared to others, suggesting a traffic of CD8 T cells from blood to tumor microenvironment under the effect of ICIs in patients with an improved response to treatment (29).

Papadaki and colleagues, assessed the PD-L1 expression on CTCs and circulating immune cells in a heterogeneous population of ES and LS-SCLC patients (106). They showed as PD-L1 expression on PBMC is associated with CTCs detection and poor patient outcome. No information about treatments received by patients included are available (106). The same working group suggested a significant negative role of post-treatment PD-L1^high^ PBMCs on OS (8.4 versus 15.7 months; p=0.007) in a small cohort of patients (N=16) receiving first line chemo-immunotherapy (107).

Peripheral immune cells have been studied as biomarkers in clinical setting comprising immune-modulation as a surrogate of the activity of the immune system against the tumor. Recently, Galindo Campos and colleagues presented the data of a small observational study including ES-SCLC patients (N=20) receiving CT plus ICIs (108). They reported an increase proportion of Ki67 expression in CD4+CXCR5+ cells, corresponding to proliferative follicular helper T cells, in ICOS+ and TIM-3 cytotoxic T cells, in peripheral blood samples of patients experiencing durable control rate to anti-PD-L1 (108). Although more data are needed in a wider cohort in RCTs, these data suggest circulating immune cells as promising biomarkers.

### Other circulating biomarkers and future perspectives

Even less data is available on the predictive role of other circulating components from ES-SCLC patients receiving ICIs.

Cytokines are soluble messenger directly involved in inflammatory process and signaling. They could have pro-inflammatory activity or an immunosuppressive behavior, influencing directly the tumor microenvironment and response to ICIs (109). Serum cytokines have been studied as predictive biomarkers in a retrospective analysis comparing SCLC patients treated with ipilimumab plus CT in the phase II ICE trial with a cohort of patients receiving CT alone (110). The authors found that patients treated with ipilimumab plus CT experienced better outcome in presence of a high serum IL-2 level at baseline compared to low. This association was not shown for patients receiving CT alone. On the contrary, IL-6 and TNF alpha were reported to be negative predictors for chemo-immunotherapy; whereas patients with an increase IL-4 level treated with ICIs, were reported to experience a better outcome (110). These results suggest cytokines evaluation as a promising field to be investigating.

MicroRNA (miRNA) are short non-coding RNA which regulates the expression levels of proteins and RNAs. A single miRNA can regulate a large number of RNA transcripts in human cells. Their aberrant expression could lead to a dysregulation of gene expression and to a pathologic behavior of cells, since they appear to be differentially expressed in normal lung and SCLC patients (111).

A recent meta-analysis showed that an increase in tumor suppressive miRNA and a decrease in oncogenic microRNA expression are associated with ES-SCLC patients prognosis (112). No association with PFS was reported in the meta-analysis. To note, every single miRNA should be investigated separately since its role on tumorigenesis and on prognosis could be different.

Circulating miRNAs have been comparatively less studied as blood-based biomarkers in SCLC but since miRNAs are stably detectable in plasma, they have a great potential. Most of the research experiences focused on the prognostic role and data on their predictive value are scarce. A recent study reported two serum miRNA, miR-92b and miR-375, as informative for assessing chemoresistance in patients with SCLC (113). A group of miRNA targeting PD-1 or PD-L1 through a network analysis have been identified (114). For the best of our knowledge, no data about the predictive role of miRNA in patient receiving immunotherapy plus platinum-based CT have been published.

The HLA is a group of membrane protein of the MHC involved in the identification of self and non-self-antigens, presenting antigenic peptides to the T-cell receptor (TCR) on T cells. Thus, HLA play an important role in the immune response in cancer patients. Garassino et al, in an exploratory analysis of the CASPIAN trial, studied the association between HLA-I/II and patients’ outcome, using a next generation sequencing approach on germline cells (115). The HLA-DQB1∗03:01 allele of the MHC class II appeared to be associated with longer OS in patients receiving durvalumab plus tremelimumab and CT (HR 0.59) but not in the durvalumab plus CT or CT arm (HR 0.93 and 0.94, respectively) (115). Even if not conclusive, data suggest a role of the antigen presenting machinery in the immunologic vulnerability of this disease and lays the foundation for further investigations.

As already presented, the molecular subtyping of SCLC establishes a new opportunity to stratified patients with different therapeutic vulnerability. However, gene expression profile appears difficult to perform in clinical practice and tumor heterogeneity is hardly catchable with a single tumor sample. In the research for a practical biomarker, molecular profiling of SCLC on liquid biopsy have been studied. The methylation of DNA is one of the most prevalent epigenetic alterations in SCLC. cfDNA- methylation profile seems to be able to discriminate between SCLC subtypes and therefore to be a possible biomarker. Heek and colleagues reported that DNA methylation can provide accurate classification of SCLC and that this approach could be applied in blood samples (95). Similarly, Chemi and colleagues showed a prognostic role of DNA-methylation level and the possibility to discriminate among SCLC subtypes (116). Sami UI Haq performed a comprehensive profilation of the methylome of SCLC patients (N=74) on cfDNA, identifying two clusters of methylation with different prognosis. Although the significance value was lost adjusting results for stage, authors stated that the study was not powered to identify stage-independent differences (117).

SCLC signature was also explored through the promoter state of cell-free chromatin in circulation through chromatin immunoprecipitation and sequencing of cell-free nucleosomes (cfChIP-seq). Fialkoff and colleagues reported a concordance of gene expression inferred from plasma cell-free chromatin and tumor transcriptome at the level of the individual patient. In particular, cfChIP-seq profiling, was able to identify activity of transcription factors, as ASCL1, NEUROD1 E POU2F3. No prognostic or predictive data have been reported, jet (118).

## Conclusion

Despite positive results obtained with chemo-immunotherapy, prognosis of SCLC patients remains dismal and only a subgroup of patients benefits from the addition of ICIs. The prospective identification of patients more likely to benefit from treatment is challenging in SCLC. In order to overcome this urgent unmet medical need, several analyses have been performed on tissue samples. However, classical predictive biomarkers, like PD-L1 and TMB, failed to demonstrate a strong predictive role. Indeed, a very low expression of PD-L1 is present on tumor cells and on immune cells; on the contrary, SCLC presents in average a high somatic burden, precluding TMB to be a reliable biomarker. The study of the tumor immune microenvironment supplies interesting data on the biology of this immune-cold disease. It is composed by several cell types whose behavior could be influenced by the surrounding signals and cytokines. New technologies play an important role in biomarker discovery since they could identify interactions between distinct cell components, critical to explain the susceptibility to treatment. CD8/MHC-I expression is particularly promising in the selection of patients. Indeed, CD8 cells are effectors of immune response to treatment and MHC-I levels mirror the deficiency in antigen presenting mechanism supposed for SCLC. However, definitively data in randomized controlled trials are needed to confirm this hypothesis. SCLC subtyping needs caution since data are not conclusive in consider inflamed gene signature as a clear predictive biomarker and the design of trials powered to demonstrate its role are urgent needed. In SCLC, due to its high intra-tumoral and inter-tumoral heterogeneity, samples obtain from a single tissue biopsy could do not catch the entire tumor profile, since tissue biopsy is only a partial photograph of the disease in a specific time and could not easily repeated during the course of treatment to capture temporal heterogeneity. Moreover, tumor samples come frequently from small biopsies with a high probability of necrotic tissue. In this context, liquid biopsy could overcome these limitations. Few data are available on its predictive role. Encouraging, although preliminary, data are available for cfDNA sequencing, cytokines analysis and HLA genotyping but deserve further investigation.

In conclusion, the identification of predictive biomarkers in ES-SCLC patients remain an unmet medical need. Every effort should be made to plan large translational study in order to understand the complex biology behind this apparently “simple” disease and RCTs powered to demonstrate the predictive role of a supposed biomarker.

## Author contributions

ML: Conceptualization, Data curation, Investigation, Methodology, Validation, Visualization, Writing – original draft, Writing – review & editing, Resources. MR: Conceptualization, Data curation, Investigation, Methodology, Resources, Validation, Visualization, Writing – original draft, Writing – review & editing. LB: Writing – review & editing, Validation. SF: Writing – review & editing, Visualization. AD: Writing – review & editing, Validation. AF: Writing – review & editing, Validation, Visualization. VG: Resources, Supervision, Validation, Writing – review & editing. GP: Conceptualization, Data curation, Funding acquisition, Investigation, Methodology, Resources, Supervision, Validation, Visualization, Writing – original draft, Writing – review & editing, Project administration.
